# Direct Medical Expenditures Associated with Eye Complications among Adults with Diabetes in the United States

**DOI:** 10.1155/2020/2864069

**Published:** 2020-05-15

**Authors:** Abdulkarim M. Meraya, Monira Alwhaibi, Moteb A. Khobrani, Hafiz A. Makeen, Saad S. Alqahtani, David Banji

**Affiliations:** ^1^Pharmacy Practice Research Unit, Department of Clinical Pharmacy, College of Pharmacy, Jazan University, Jazan, Saudi Arabia; ^2^Department of Clinical Pharmacy, College of Pharmacy, King Saud University, Riyadh, Saudi Arabia; ^3^Medication Safety Research Chair, College of Pharmacy, King Saud University, Riyadh, Saudi Arabia; ^4^Department of Clinical Pharmacy, College of Pharmacy, King Khalid University, Abha, Saudi Arabia

## Abstract

**Objectives:**

National estimates of healthcare expenditures by types of services for adults with comorbid diabetes and eye complications (ECs) are scarce. Therefore, the first objective of this study is to estimate total healthcare expenditures and expenditures by types of services (inpatient, outpatient, prescription, and emergency) for adults with ECs. The second objective is to estimate the out-of-pocket spending burden among adults with ECs. *Study Design*. A cross-sectional study design using data from multiple panels (2009-2015) of the Medical Expenditure Panel Survey was employed. The sample included adults aged 21 years or older with diabetes (*n* = 8,420). *Principal Findings*. Of adults with diabetes, 18.9% had ECs. Adults ECs had significantly higher incremental total medical expenditures of $3,125. The highest incremental expenditures were associated with outpatient and prescription drugs. After controlling for sex, age, race, poverty level, insurance coverage, prescription coverage, perceived physical and mental health, the number of chronic physical and mental conditions, marital status, education, the region of residence, smoking status, exercise, and chronic kidney disease (CKD), there was no difference in the out-of-pocket spending burden between adults with and those without ECs. However, adults with comorbid diabetes and CKD were more likely to have the out-of-pocket spending burden than those without CKD.

**Conclusions:**

The study showed that ECs in individuals with diabetes are associated with high incremental direct medical and out-of-pocket expenditures. Therefore, it requires more health initiatives, interventions, strategies, and programs to address and minimize the risk involved in such affected individuals.

## 1. Introduction

Diabetes, a metabolic disorder, impairs health-related quality of life (HRQoL) and exerts a substantial socioeconomic burden, not only on the individuals but also on the family and the society at large. Incidents of diabetes are on the constant rise, so are the associated expenditures. The prevalence of diabetes has increased many folds in the last fifty years, and currently, 40% of the citizens of the United States of America (US), aged 65 years and above, are with diabetes. The prevalence of diabetes was 463 million adults (20-79 years) in 2019 and is projected to rise to 700 million in 2045 worldwide and would exponentially grow from 48 million in 2019 to 56 million in 2030 and 63 million in 2045 in the US alone, which is equivalent to one in eight adults with diabetes and one in six adults at potential risk of type 2 diabetes, consuming 43% of global diabetes-related health expenditures in the US in 2019. Besides, it is estimated that 38.1% of adults (20-79 years) afflicted with diabetes were undiagnosed in the US [1, 2].

Uncontrolled diabetes compromises HRQoL and may increase mortality risk due to both the microvascular (stroke, coronary heart diseases, peripheral vascular diseases, etc.) and macrovascular (nephropathy, retinopathy, neuropathy, etc.) complications, and death attributable to diabetes is 1.2 million in 2019. Diabetes, therefore, is also considered a principal cause of end-stage kidney disease, adding nearly 44% of new cases every year [3]. Around 19% of adults with diabetes had eye complications (ECs) between 1999 and 2006 [4], and about 4% of adults aged above 40 years with diabetes had advanced diabetic retinopathy associated with macular edema and proliferative diabetic retinopathy that resulted in severe vision loss. Diabetic eye complications are the prime cause for vision loss, and according to two population-based studies, 2.6 million people were visually impaired because of diabetes in 2015, and it is projected to rise to 3.2 million in 2020 [5, 6]. Diabetes-related ECs affect the psychological well-being and HRQoL and impact the economy of the individuals. The financial burden of diabetes-related ECs is found to be high among adults in the US, particularly those with any degree of the eye or renal complications [7]. The increased direct medical expenditures associated with ECs impose an economic burden on both individuals and payers.

Brook et al. reported that both diabetic macular edema and diabetic retinopathy are associated with higher direct medical cost and absenteeism [8]. Nevertheless, a comparison of different types of medical expenditures using US national data among individuals with comorbid diabetes and ECs is scarce. Also, studies on the out-of-pocket spending among this specified individual group are limited. Therefore, we aimed to examine the incremental burden of ECs on total and out-of-pocket spending among adults with diabetes in the US.

## 2. Materials and Methods

### 2.1. Study Design

The study was a retrospective cross-sectional design. We used data extracted from multiple panels (2009–2015) of the US Medical Expenditure Panel Survey (MEPS).

### 2.2. Data Source

MEPS is a nationally representative survey conducted by the Agency for Healthcare Research and Quality (AHRQ) of the US noninstitutionalized civilian population. MEPS contains questionnaire responses of deidentified noninstitutionalized persons and their families, their medical providers, and employers in the US. MEPS permits the weighting of the data to provide nationally representative estimates of the US noninstitutionalized civilian population [9]. We used households, diabetes care surveys, and medical condition files from the MEPS. Data from MEPS was collected in the period between 2009 and 2015. This more comprehensive duration was considered to increase the sample size. Based on the recommendations of the AHRQ, we used alternate years, 2009, 2011, 2013, and 2015, to avoid the repetition of data of the same participant [10]. Information regarding surveyed patients' mental and physical health, demographic and socioeconomic characteristics, employment, access to care, medical care expenditures, types of medical care expenditures, and satisfaction with healthcare were extracted from the household component of the survey [9]. Medical conditions reported by the participants, which were available in either the household file or the medical condition file, were used.

### 2.3. Study Sample

**Figure 1:**
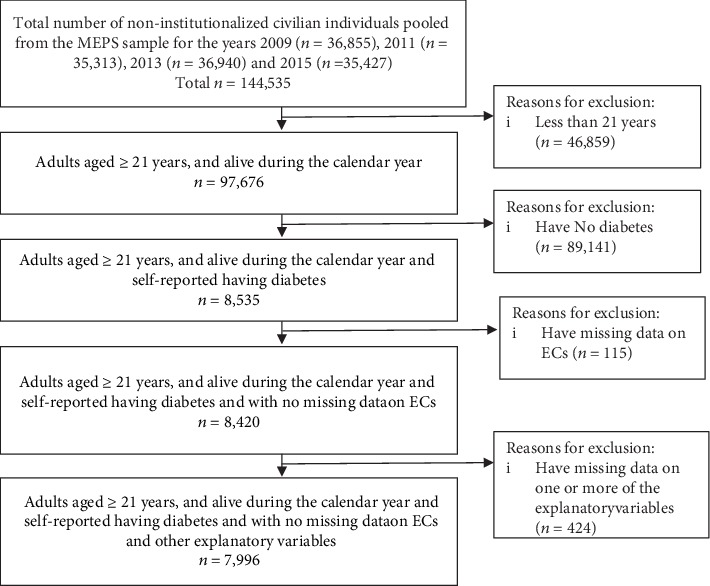
Flow diagram of the study sample.

The study sample consists of adults aged 21 years or older who had diabetes. In the MEPS, these samples correspond to the individuals with diabetes who had responded positively to a question “Have you ever been told by a doctor or health professional that you have diabetes?” Later on, who participated in a diabetes care survey to gather information related to diabetes management, diabetes-related complications, and recommended preventive care.  Figure 1 displays the flow diagram of the study sample.

### 2.4. Measures

#### 2.4.1. Outcomes

*(1) Healthcare Expenditures*. Healthcare expenditures are defined as the sum of direct payments for care provided during the year, according to MEPS. In the MEPS, healthcare expenditures include all payments for all healthcare purposes and not those only related to diabetes or other chronic conditions. The direct payments include twelve sources of payment categories such as out-of-pocket by patients or families, Medicare, Medicaid, private insurance, Veteran Administration, and worker's compensation. Total annual per-person healthcare expenditures were calculated as the sum of inpatient, outpatient, emergency, dental, home health, vision, prescription drugs, and other medical supplies. Additional services included other medical equipment utilized, such as expenditures for ambulance services, orthopedic items, hearing devices, prostheses, bathroom aids, medical equipment, disposable supplies, alterations and modifications, and other miscellaneous items or services that were obtained, purchased, or rented during the year. All expenditures were inflation-adjusted to 2015 US dollars (USD) using the consumer price index for medical services from the Bureau of Medical Services.

*(2) Out-of-Pocket Spending Burden*. Out-of-pocket expenditures included annual deductibles, copayments, and coinsurance for services and payments for services that were not covered by health insurance. The out-of-pocket spending burden was calculated as the ratio of out-of-pocket healthcare expenditures to personal income and expressed as a percentage, with the out-of-pocket burden varying from 0 to 100. Following previous studies [11, 12], we imposed $100 of personal income for individuals with less than $100 personal income. This approach affected only 11% of the sample and did not change the final estimates. Based on the published literature, a high out-of-pocket spending burden was defined as spending 10% or more of personal income on healthcare [13, 14].

### 2.5. Key Explanatory Variable

#### 2.5.1. ECs (Yes/No)

An affirmative answer to the question “Has your diabetes caused problems with your eyes that needed to be treated by an ophthalmologist?” was confirmed as EC.

### 2.6. Other Explanatory Variables

Other explanatory variables include sex (female, male); age (21-39, 40-49, 50-64, and 65 years or older); race/ethnicity (White, African American, Latino, and others); marital status (married, separated/divorced, widowed, and never married); state of poverty: poor (less than 100% federal poverty line), near poor (100% to less than 200%), middle income (200% to less than 400%), and high income (greater than or equal to 400%); health insurance coverage (private, public, and uninsured); prescription drug coverage (yes or no); presence of other cooccurring physical conditions (asthma, arthritis, cancer, gastroesophageal reflux disease (GERD), heart diseases, hypertension, osteoporosis, thyroid, and chronic obstructive pulmonary disease (COPD)); presence of other cooccurring mental conditions (anxiety and/or depression); presence of CKD (yes/no: a positive response to the question, “Has your diabetes caused problems with your kidneys?”); perceived physical and psychological health; smoking status (current smoker and others); physical activity (vigorous or moderate (activities at least three days a week) and others); and region of residence (Northwest, Midwest, South, and West).

### 2.7. Ethical Approval and Consent

The current study was based on a publicly available dataset, MEPS, and there was no direct contact with survey participants. Hence, ethical approval was not applicable.

### 2.8. Statistical Techniques

#### 2.8.1. Generalized Linear Model

Generalized linear model (GLM) regressions with a logarithmic link were used to estimate total medical expenditure as well as medical expenditure by service type. GLM is an attractive alternative to OLS regressions on log-transformed expenses because it corrects for heteroscedasticity and avoids retransformation bias [15, 16]. Therefore, in this study, we used GLM with log link and gamma family distribution to estimate the adjusted medical expenditures associated with CKD and ECs.

#### 2.8.2. Two-Part Regression Model

Two-part logit-generalized linear regression models were used as a secondary analysis to estimate total medical expenditures and medical expenditures by service type. This model has been widely used in situations where there is a large number of nonusers of health services or there are excess zeros in resource use or cost data [16]. Two-part regression models are useful when there are many adults in the sample with zero expenditure. For example, not all adults with diabetes use inpatient services. Therefore, those adults would have zero inpatient expenditures. In the two-part regression models, the first part was a logistic regression estimating the probability of positive expenditures. In the second part, a GLM with a logarithmic link and gamma distribution was used to estimate medical expenditures in the subsample with positive expenditures as derived from the logistic regression. The expenditures are then obtained by multiplying the predictions from the two parts.

We have used recycled prediction [17] to estimate adjusted means for adults with ECs and those without ECs and to estimate excess healthcare expenditures attributable to ECs among adults with diabetes. In all recycled prediction models, confidence intervals were obtained using 2000 bootstrap replications using the percentile method.

#### 2.8.3. Multivariable Logistic Regression

Logistic regression was used to assess the relationships between the ECs and the out-of-pocket spending burden.

In all adjusted models, the following variables are controlled for: sex, age, race/ethnicity, marital status, poverty status, health insurance coverage, prescription drug coverage, the presence of other coccurring physical or mental conditions, CKD, perceived physical and mental health, smoking status, and physical activity. All analyses were conducted using survey procedures in the Statistical Analysis System (SAS®) version 9.4 and STATA 15.1. Diabetes care survey weights were used in the analyses. These weights adjust for diabetes care survey nonresponse, and the sum of these weights is equal to the number of individuals with diabetes in the US civilian noninstitutionalized population in a given year [18]. Diabetes care survey weights were divided by four as the sample was pooled from four years to estimate the annualize weighted numbers following the recommendations of MEPS [19] and other studies [20, 21].

## 3. Results

### 3.1. Description of the Study Sample

Table 1 summarizes the characteristics of the studied adults with diabetes. The study sample consisted of 8,420 diabetic adults; out of them, 18.9% had ECs and 11.2% had CKD. Most of the study sample were females (50.7%), white (61.3%), and married (57.4%). Additionally, most of the study sample had one additional chronic physical condition or more (88.2%). Sex, race, poverty status, insurance coverage, prescription drug coverage, perceived physical and mental health, the number of mental and physical health conditions, marital status, education, the region of residence, and CKD were associated with ECs in the bivariate analysis.

### 3.2. Unadjusted Direct Medical Expenditures

Table 2 displays the unadjusted mean expenditures for pooled samples of adults with diabetes and ECs. Adults with ECs have significantly higher total medical expenditures ($19,921 (95% CI: $16,832–$23,010)) than those without ECs ($11,585 (95% CI: $11,016–$12,155)). The major cost drivers for adults with ECs were prescription ($6,410), outpatient ($5,771), and inpatient ($5,551) expenditures. The differences in expenditures between adults with ECs and those without were significant for all types.

### 3.3. Adjusted Direct Medical Expenditures

Table 3 displays the adjusted mean expenditures by types of services from adjusted GLM with log link and two-part regression models. After controlling for sex, age, race, poverty level, insurance coverage, prescription coverage, perceived physical and mental health, the number of chronic physical and mental conditions, marital status, education, the region of residence, smoking status, exercise, and CKD, adults with ECs had higher incremental total medical expenditures ($3,154 (95% CI: $3,110–$3,203)) relative to those without ECs. ECs were significantly associated with higher incremental outpatient ($992 (95% CI: $976–$1,007)), prescription ($1,173 (95% CI: $1,156–$1,190)), other ($99 (95% CI: $96–$103)), and out-of-pocket ($155 (95% CI: $154–$157)) expenditures relative to those without ECs.

As shown in Table 4, Hispanics had significantly lower incremental total expenditures than white adults. Furthermore, uninsured adults had lower incremental total medical expenditures than those with private insurance. Similarly, adults without prescription drug coverage also had lower incremental total medical expenditure. About physical health, incremental total medical expenditures increase as the number of chronic physical conditions increases. Additionally, adults who perceived their physical health as good or fair/poor had higher incremental total medical expenditures than those who perceived their physical health as excellent/very good.

In the secondary analyses that used a two-part model to account for zero expenditure (Table 3), ECs were associated with higher incremental direct medical expenditure, and the estimates were very similar to the estimated expenditure by GLM models.

### 3.4. Out-of-Pocket Healthcare Spending Burden

Among adults with diabetes, 22.4% spent 10% or more of their income on healthcare. A higher rate of adults with ECs had a high out-of-pocket spending burden compared with those without ECs (27.0% vs. 21.2%). Also, a large percentage of adults with CKD had a high out-of-pocket spending burden compared with those without CKD (33.2% vs. 21.1%).

In multivariable logistic regression, after controlling for sex, age, race, poverty level, insurance coverage, prescription coverage, perceived physical and mental health, the number of chronic physical and mental conditions, marital status, education, the region of residence, smoking status, exercise, and ECs, adults with CKD were more likely to have a high out-of-pocket healthcare spending burden than adults without CKD (OR: 1.409 (95% CI: 1.096–1.810); *P* value = 0.008). However, there was no difference in the out-of-pocket healthcare spending burden between adults with ECs and those without ECs after controlling for demographic and clinical factors (OR: 1.027 (95% CI: 0.842–1.252); *P* value = 0.795).

Men were less likely to have a high out-of-pocket spending burden than women. Likewise, African Americans, Hispanics, and other races were less likely to have high out-of-pocket spending burdens than white adults. However, adults with a higher number of chronic physical conditions are more likely to have a high out-of-pocket spending burden. Similarly, uninsured adults were more likely to have a high out-of-pocket spending burden.  Table 5 shows adjusted odds ratios and their 95% confidence intervals for demographic, clinical, and socioeconomic factors from logistic regression on a high out-of-pocket spending burden.

## 4. Discussion

The study used the MEPS to estimate the incremental direct medical expenditures associated with ECs among adults with diabetes in the US. Among adults with diabetes, the estimated weighted number of those with ECs from the pooled sample was 4,206,671; and the estimated mean direct medical expenditures were $3,154. Therefore, the adjusted financial burden of ECs was $13.3 billion per year among adults with diabetes in the US. Diabetes is associated with high medical expenditures. In 2017, diabetes was associated with $237 billion in direct medical spending [22]. Our study findings corroborate with other studies regarding the large proportion of these expenditures being associated with diabetes-related complications [7, 8, 23]. In the US, preventive programs and initiatives are required among adults with diabetes to minimize the risk of diabetes-related complications and to curb the high direct and indirect healthcare expenditures among adults with diabetes.

Our results suggested higher healthcare expenditures among US adults with comorbid diabetes and ECs as compared to other Asian and European countries [24, 25]. For instance, a study in Singapore estimated the direct medical expenditures associated with ECs among adults with diabetes to be $2,219.4 [24], which was lower than our estimates. Furthermore, a previous study indicated that the US has the highest healthcare expenditures for diabetes [26]. The differences in healthcare expenditures could be due to the lower prevalence of diabetes and diabetes-related complications in these countries [26, 27]. In general, the US has the highest per capita health spending in the world [28]. The economic impact of diabetic ECs is a definite contributor to the overall HRQoL burden. Diabetic EC is a common microvascular complication leading to the cause of irreversible loss of vision among diabetic adults. Health programs and initiatives are required to screen for diabetes at the early stages and early detection of diabetes-related complications.

Our results showed that uninsured adults with diabetes had lower direct medical expenditures than insured adults. This finding was consistent with previously published reports indicating that uninsured adults had lower medical expenditures than insured adults in the US [29, 30] because they may have lower access to medical care [31]. On the contrary, they may face a high economic burden in terms of out-of-pocket spending. Our results support the finding that uninsured adults were more likely to have a high out-of-pocket spending burden. Similarly, another published study showed that uninsured adults in the US have higher out-of-pocket spending than those with private or public insurance [32].

This study has multiple strengths; first, it used nationally representative data with high generalizability on US adults with diabetes. Also, the analyses have adjusted for many covariates that can affect healthcare expenditure. Furthermore, the robustness of the relationship between diabetes-related complications and healthcare expenditures was examined using various models that account for zero expenditure and skewed distribution of expenditures. However, these findings should be interpreted in the context of some limitations. The limitations of the study are as follows: all information were self-reported and were subject to recall bias; we did not differentiate between type 1 and type 2 diabetes in the analyses, as this information is not available in MEPS. Also, information on other diabetes-related complications rather than CKD and ECs were not available in the MEPS. A positive response to the question “Has your diabetes caused problems with your eyes that needed to be treated by an ophthalmologist?” was used to identify adults with ECs. Therefore, we were not able to capture adults with ECs who did not see an ophthalmologist or were not offered treatment. Finally, due to the nature of the study design, it is not possible to build a causal relationship between the factors under study and the outcomes.

The findings of this study showed that ECs are associated with high incremental direct medical expenditure. Additionally, ECs were associated with high out-of-pocket expenditures, incremental inpatient, and prescription expenditures. Therefore, health initiatives and programs are required to reduce the development of diabetes and diabetes-related complication. Interventions and strategies need to address and minimize the risk of hospitalizations and emergency department visits among adults with comorbid ECs and diabetes.

## Figures and Tables

**Table 1:** Descriptive statistics of adults with diabetes (*n* = 8,420). Row Wt.%. Medical Expenditure Panel Survey (2009, 2011, 2013, and 2015).

	All (Wt.%)	Eye complications	No eye complications	*P* value
All	100%	18.9	81.1	
Sex				0.041
Women	50.7	19.9	80.1	
Men	49.3	17.8	82.2	
Age				0.100
21-39	6.5	16.3	83.7	
40-49	12.9	17.2	82.8	
50-64	37.9	18.4	81.6	
65+	42.7	20.2	79.8	
Race				<0.001
White	61.3	16.2	83.8	
African American	15.4	24.2	75.8	
Hispanic	15.2	25.2	74.8	
Others	8.1	17.3	82.7	
Poverty status				<0.001
Poor	14.4	25.7	74.3	
Near poor	22.2	22.8	77.2	
Middle income	31.0	18.6	81.4	
High income	32.4	13.5	86.5	
Health insurance				<0.001
Private	58.2	15.3	84.7	
Public	34.4	25.0	75.0	
Uninsured	7.5	19.0	81.0	
Prescription drug coverage				<0.001
Yes	92.2	19.5	80.5	
No	7.8	11.5	88.5	
Perceived physical health				<0.001
Excellent/very good	26.8	12.7	87.3	
Good	39.1	15.1	84.9	
Fair/poor	34.1	28.2	71.8	
Number of chronic physical conditions				<0.001
No physical condition	11.8	12.7	87.3	
1-2	48.7	17.3	82.7	
3-4	30.8	21.8	78.2	
≥5	8.7	25.9	74.1	
Perceived mental health				<0.001
Excellent/very good	50.0	14.2	85.8	
Good	34.6	21.8	78.2	
Fair/poor	15.3	27.9	72.1	
Number of chronic mental conditions				0.002
No mental chronic condition	86.2	18.2	81.8	
≥1	13.8	23.5	76.5	
Marital status				0.014
Married	57.4	17.6	82.4	
Widow	13.7	21.9	78.1	
Separated/divorced	18.1	20.7	79.3	
Never married	10.8	19.0	81.0	
Education				<0.001
Less than high school	20.8	24.3	75.7	
High school	34.0	19.8	80.2	
Greater than high school	45.1	15.8	84.2	
Region of residence				0.031
Northeast	16.99	20.8	79.2	
Midwest	21.52	16.3	83.7	
South	41.61	20.2	79.8	
West	19.88	17.4	82.6	
Current smoker				0.093
Yes	14.6	21.1	78.9	
No	85.4	18.5	81.5	
Heavy physical exercise				
Yes	36.7	15.4	84.6	<0.001
No	63.3	20.9	79.1	
CKD				
Yes	11.2	49.1	50.9	<0.001
No	88.8	15.0	85.0	

Note: based on 8,420 adults aged 21 years or older, alive during the calendar years, and reported having diabetes. The *P* values were derived from the chi-squared tests between groups of adults with complications and explanatory variables. Physical chronic conditions included asthma, arthritis, cancer, gastroesophageal reflux disease, heart diseases, hypertension, osteoporosis, thyroid, and chronic obstructive pulmonary disease. Mental chronic conditions included anxiety and/or depression. Missing data for the explanatory variables are not presented. Wt.: weighted.

**Table 2:** Unadjusted total and incremental average annual healthcare expenditures (2015 US dollars) among adults with diabetes by the presence of eye complications (*n* = 8,420). Medical Expenditure Panel Survey (2009, 2011, 2013, and 2015).

Expenditures	Eye complications		No eye complications		Incremental difference		*P* value
	Mean ($)	95% CI	Mean ($)	95% CI	Mean ($)	95% CI	
Total	$19,921	($16,832–$23,010)	$11,585	($11,016–$12,155)	$8,335	($5,169–$11,502)	<0.001
Inpatient	$5,551	($4,425–$6,677)	$3,006	($2,613–$3,399)	$2,545	($1,366–$3,723)	<0.001
Outpatient	$5,771	($4,698–$6,843)	$3,328	($3,098–$3,558)	$2,442	($1,322–$3,563)	<0.001
Prescription	$6,410	($5,032–$7,789)	$3,927	($3,692–$4,163)	$2,483	($1,072–$3,894)	0.001
Emergency	$504	($416–$591)	$351	($306–$396)	$153	($53–$252)	0.003
Others	$264	($158–$371)	$99	($80–$117)	$166	($58–$274)	0.003
Out-of-pocket	$1,568	($1,354–$1,782)	$1,314	($1,243–$1,385)	$254	($25–$484)	0.030

Note: based on 8,420 adults aged 21 years or older, alive during the calendar years, and reported having diabetes.

**Table 3:** Total and incremental average annual healthcare expenditures (2015 US dollars) among adults with diabetes by the presence of eye complications (*n* = 8,420). Medical Expenditure Panel Survey (2009, 2011, 2013, and 2015).

	Eye complications		No eye complications		Incremental difference		*P* value
	Mean ($)	95% CI	Mean ($)	95% CI	Mean ($)	95% CI	
Adjusted generalized linear model with log link							
Expenditures							
Total	$14,895	($14,687–$15,123)	$11,741	($11,577–$11,921)	$3,154	($3,110–$3,203)	<0.001
Inpatient	$4,460	($4,361–$4,558)	$3,485	($3,408–$3,562)	$975	($953–$996)	0.082
Outpatient	$4,168	($4,103–$4,233)	$3,176	($3,127–$3,225)	$992	($976–$1,007)	<0.001
Prescription	$5,003	($4,929–$5,074)	$3,830	($3,773–$3,884)	$1,173	($1,156–$1,190)	<0.001
Emergency	$494	($484–$504)	$388	($381–$396)	$106	($104–$108)	0.098
Others	$231	($222–$241)	$132	($127–$137)	$99	($96–$103)	0.001
Out-of-pocket	$1,297	($1,282–$1,312)	$1,142	($1,129–$1,155)	$155	($154–$157)	0.036

Adjusted two-part regression model							
Expenditures							
Total	$13,363	($13,182–$13,548)	$10,563	($10,419–$10,710)	$2,800	($2,763–$2,838)	<0.001
Inpatient	$4,041	($3,969–$4,108)	$3,209	($3,149–$3,266)	$832	($820–$843)	0.971
Outpatient	$4,105	($4,048–$4,165)	$3,180	($3,134–$3,226)	$926	($913–$938)	0.001
Prescription	$4,437	($4,382–$4,490)	$3,404	($3,362–$3,445)	$1,033	($1,020–$1,045)	<0.001
Emergency	$494	($484–$503)	$389	($381–$396)	$105	($103–$107)	0.275
Others	$215	($208–$223)	$123	($119–$128)	$92	($89–$95)	0.027
Out-of-pocket	$1,297	($1,282–$1,313)	$1,142	($1,129–$1,155)	$155	($154–$157)	0.038

Note: based on 8 ,420 adults aged 21 years or older, alive during the calendar years, and reported having diabetes. Adjusted models included sex, age, race/ethnicity, marital status, poverty status, health insurance coverage, drug prescription coverage, number of mental and physical health conditions, smoking status, geographic area of residence, and physical activity.

**Table 4:** Adjusted mean total direct medical expenditure for eye complications (2015 US$). Medical Expenditures Panel Survey (2009, 2011, 2013, and 2015).

	Adjusted Mean^a^	95% CI	*P* value
Eye complications			
No eye complications (Ref.)			
Eye complications	$3,325	($1,799–$4,852)	<0.001
CKD			
No CKD (Ref.)			
CKD	$7,964	($5,332–$10,596)	<0.001
Sex			
Women (Ref.)			
Men	$725	(-$347–$1,798)	0.184
Age			
21-39 (Ref.)			
40-49	-$585	(-$3,354–$2,185)	0.678
50-64	$820	(-$1,723–$3,364)	0.526
65+	$595	(-$2,103–$3,294)	0.664
Race			
White (Ref.)			
African American	-$844	(-$2,124–$436)	0.195
Hispanic	-$2,267	(-$3,892–-$642)	0.006
Others	-$2,155	(-$4,467–$157)	0.067
Poverty status			
Poor (Ref.)			
Near poor	-$1,839	(-$3,854–$177)	0.074
Middle income	-$1,988	(-$4,083–$107)	0.063
High income	-$1,984	(-$4,317–$349)	0.095
Health insurance			
Private (Ref.)			
Public	$18	(-$1,632–$1,669)	0.983
Uninsured	-$7,485	(-$8,816–-$6,154)	<0.001
Prescription drug coverage			
Yes (Ref.)			
No	-$1,929	(-$3,424–-$435)	0.012
Perceived physical health			
Excellent/very good (Ref.)			
Good	$2,229	($1,063–$3,395)	<0.001
Fair/poor	$6,378	($4,812–$7,944)	<0.001
Number of chronic physical conditions			
No physical condition (Ref.)			
1-2	$5,038	($4,173–$5,902)	<0.001
3-4	$9,985	($8,637–$11,333)	<0.001
≥5	$14,546	($12,240–$16,852)	<0.001
Perceived mental health			
Excellent/very good (Ref.)			
Good	-$603	(-$1,680–473)	0.27
Fair/poor	$1,118	(-$418–$2,655)	0.153
Marital status			
Married (Ref.)			
Widow	$1,476	(-$72–$3,025)	0.062
Separated/divorced	-$153	(-$1,631–$1,325)	0.838
Never married	$420	(-$1,127–$1,967)	0.593
Education			
Less than high school (Ref.)			
High school	-$46	(-$1,455–$1,363)	0.949
Greater than high school	$1,782	($226–$3,337)	0.025
Region of residence			
Northeast (Ref.)			
Midwest	$335	(-$1,676–$2,345)	0.743
South	-$1,694	(-$3,239–-$149)	0.032
West	-$1843	(-$3,533–-$154)	0.033
Current smoker			
Yes (Ref.)			
No	$900	(-$318–$2,118)	0.147
Heavy physical exercise			
Yes (Ref.)			
No	$1,676	($595–$2,757)	0.003

Note: based on 8,420 adults aged 21 years or older, alive during the calendar years, and reported having diabetes. CKD: chronic kidney disease; CI: confidence interval. ^a^Marginal effects based on a generalized linear regression model.

**Table 5:** Weighted row percentages, adjusted odds ratios, and 95% confidence for demographic, clinical, and socioeconomic factors from logistic regression on a high out-of-pocket spending burden. Medical Expenditure Panel Survey (2009, 2011, 2013, and 2015).

	All (%)	AOR (95% CI)	*P* value
Eye complications			
No	21.1	Reference	
Yes	27.0	1.027 (0.842–1.252)	0.795
Chronic kidney disease			
No	21.0	Reference	
Yes	33.2	1.409 (1.096–1.81)	0.008
Sex			
Women	27.2	Reference	
Men	17.4	0.567 (0.486–0.662)	<0.001
Age			
21-39	20.0	Reference	
40-49	19.7	0.961 (0.634–1.457)	0.852
50-64	24.0	1.24 (0.882–1.743)	0.214
65+	22.1	1.15 (0.784–1.686)	0.472
Race			
White	23.6	Reference	
African American	19.0	0.65 (0.55–0.768)	<0.001
Hispanic	22.5	0.635 (0.515–0.785)	<0.001
Others	19.1	0.739 (0.562–0.971)	0.030
Poverty status			
Poor	39.8	Reference	
Near poor	28.2	0.518 (0.429–0.624)	<0.001
Middle income	21.2	0.301 (0.238–0.379)	<0.001
High income	11.8	0.136 (0.101–0.183)	<0.001
Health insurance			
Private	19.2	Reference	
Public	24.6	0.869 (0.679–1.111)	0.261
Uninsured	37.0	2.341 (1.632–3.36)	<0.001
Prescription drug coverage			
No	14.0	Reference	
Yes	23.1	0.88 (0.683–1.134)	0.322
Perceived physical health			
Excellent/very good	15.8	Reference	
Good	19.1	1.009 (0.825–1.234)	0.930
Fair/poor	31.2	1.413 (1.125–1.776)	0.003
Number of chronic physical conditions			
No physical condition	12.5	Reference	
1-2	20.6	1.763 (1.281–2.426)	0.001
3-4	25.3	2.01 (1.408–2.87)	<0.001
≥5	35.3	2.733 (1.761–4.24)	<0.001
Perceived mental health			
Excellent/very good	18.7	Reference	
Good	23.7	1.002 (0.823–1.22)	0.983
Fair/poor	31.4	1.159 (0.909–1.478)	0.232
Number of chronic mental conditions			
No mental chronic condition	21.3	Reference	
≥1	29.3	1.127 (0.911–1.393)	0.269
Marital status			
Married	24.7	Reference	
Widow	22.9	0.518 (0.413–0.648)	<0.001
Separated/divorced	17.6	0.349 (0.281–0.433)	<0.001
Never married	17.0	0.402 (0.298–0.542)	<0.001
Education			
Less than high school	27.2	Reference	
High school	23.0	0.965 (0.784–1.188)	0.738
Greater than high school	19.7	1.04 (0.861–1.257)	0.681
Region of residence			
Northeast	19.9	Reference	
Midwest	22.6	1.057 (0.839–1.332)	0.636
South	24.1	1.146 (0.916–1.435)	0.231
West	20.4	1.02 (0.788–1.321)	0.880
Current smoker			
Yes	23.1	Reference	
No	22.2	1.166 (0.949–1.433)	0.144
Heavy physical exercise			
Yes	17.9	Reference	
No	25.0	1.186 (0.997–1.411)	0.054

Note: based on 8,420 adults aged 21 years or older, alive during the calendar years, and reported having diabetes.

## Data Availability

The dataset supporting the conclusions of this article is available from the Medical Expenditure Panel Survey (MEPS) database and openly made available for researchers at the following website: https://meps.ahrq.gov/data_stats/download_data_files.jsp.

## Supplementary Materials

Supplementary Materials Adjusted mean total direct medical expenditure for chronic kidney disease and eye complications (2015 US$).

## References

[1] International Diabetic Federation (2019). *Diabetes Atlas, Global Fact Sheet*.

[2] Guariguata L., Whiting D. R., Hambleton I., Beagley J., Linnenkamp U., Shaw J. E. (2014). Global estimates of diabetes prevalence for 2013 and projections for 2035. *Diabetes research and clinical practice*.

[3] Duan J., Wang C., Liu D. (2019). Prevalence and risk factors of chronic kidney disease and diabetic kidney disease in Chinese rural residents: a cross-sectional survey. *Scientific Reports*.

[4] Deshpande A. D., Harris-Hayes M., Schootman M. (2008). Epidemiology of diabetes and diabetes-related complications. *Physical therapy*.

[5] Leasher J. L., Bourne R. R., Flaxman S. R. (2016). Global estimates on the number of people blind or visually impaired by diabetic retinopathy: a meta-analysis from 1990 to 2010. *Diabetes Care*.

[6] Flaxman S. R., Bourne R. R. A., Resnikoff S. (2017). Global causes of blindness and distance vision impairment 1990–2020: a systematic review and meta-analysis. *The Lancet Global Health*.

[7] Li R., Bilik D., Brown M. B. (2013). Medical costs associated with type 2 diabetes complications and comorbidities. *The American journal of managed care*.

[8] Brook R. A., Kleinman N. L., Patel S., Smeeding J. E., Beren I. A., Turpcu A. (2015). United States comparative costs and absenteeism of diabetic ophthalmic conditions. *Postgraduate medicine*.

[9] Medical Expenditure Panel Survey *U.S. Department of Human and Health Services; Agency for Healthcare Research and Quality*.

[10] Sommers J. P. (2006). An examination of state estimates using multiple years of data from the medical expenditure panel survey, household component: Agency for Healthcare Research and Quality.

[11] Gotanda H., Jha A. K., Kominski G. F., Tsugawa Y. (2020). Out-of-pocket spending and financial burden among low income adults after Medicaid expansions in the United States: quasi-experimental difference-in-difference study. *BMJ*.

[12] Banthin J. S., Bernard D. M. (2006). Changes in financial burdens for health care: national estimates for the population younger than 65 years, 1996 to 2003. *Journal of the American Medical Association*.

[13] Meraya A. M., Raval A. D., Sambamoorthi U. (2015). chronic condition combinations and health care expenditures and out-of-pocket spending burden among adults, Medical Expenditure Panel Survey, 2009 and 2011. *Preventing chronic disease*.

[14] Paez K. A., Zhao L., Hwang W. (2009). Rising out-of-pocket spending for chronic conditions: a ten-year trend. *Health affairs*.

[15] Gregori D., Petrinco M., Bo S., Desideri A., Merletti F., Pagano E. (2011). Regression models for analyzing costs and their determinants in health care: an introductory review. *International Journal for Quality in Health Care*.

[16] Mihaylova B., Briggs A., O'Hagan A., Thompson S. G. (2011). Review of statistical methods for analysing healthcare resources and costs. *Health economics*.

[17] Basu A., Rathouz P. J. (2004). Estimating marginal and incremental effects on health outcomes using flexible link and variance function models. *Biostatistics*.

[18] Agency for Healthcare Research and Quality Center (2017). Medical Expenditure Panel Survey HC-181. *2015 Full Year*.

[19] Agency for Healthcare Research and Quality Center (2009). Medical Expenditure Panel Survey HC-036. *1996-2007 Pooled Estimation File*.

[20] Ozieh M. N., Bishu K. G., Dismuke C. E., Egede L. E. (2017). Trends in healthcare expenditure in United States adults with chronic kidney disease: 2002–2011. *BMC health services research*.

[21] Coughlan D., Yeh S. T., O’Neill C., Frick K. D. (2014). Evaluating direct medical expenditures estimation methods of adults using the medical expenditure panel survey: an example focusing on head and neck cancer. *Value in Health*.

[22] American Diabetes Association (2018). Economic costs of diabetes in the U.S. in 2017. *Diabetes Care*.

[23] Ozieh M. N., Dismuke C. E., Lynch C. P., Egede L. E. (2015). Medical care expenditures associated with chronic kidney disease in adults with diabetes: United States 2011. *Diabetes research and clinical practice*.

[24] Zhang X., Low S., Kumari N. (2017). Direct medical cost associated with diabetic retinopathy severity in type 2 diabetes in Singapore. *PloS one*.

[25] Schmitt-Koopmann I., Schwenkglenks M., Spinas G. A., Szucs T. D. (2004). Direct medical costs of type 2 diabetes and its complications in Switzerland. *The European Journal of Public Health*.

[26] Zhang P., Zhang X., Brown J. (2010). Global healthcare expenditure on diabetes for 2010 and 2030. *Diabetes research and clinical practice*.

[27] Lee R., Wong T. Y., Sabanayagam C. (2015). Epidemiology of diabetic retinopathy, diabetic macular edema and related vision loss. *Eye and vision*.

[28] Reinhardt U. E., Hussey P. S., Anderson G. F. (2004). U.S. health care spending in an international context. *Health Affairs*.

[29] Agency for Healthcare Research and Quality (2020). Number of people in thousands, United States, 1996-2017. *Medical Expenditure Panel Survey*.

[30] Garfield R., Orgera K., Damico A. (2019). The uninsured and the ACA: a primer-key facts about health insurance and the uninsured amidst changes to the affordable care act. *Kaiser Family Foundation*.

[31] Coleman S., Kellerman A. (2003). *Hidden Costs, Value Lost: Uninsurance in America*.

[32] Roemer M. I. (2017). *Out-of-Pocket Health Care Expenses for Medical Services, by Insurance Coverage, 2000-2014*.

