# Supplementary material for: Direct Medical Expenditures Associated with Eye Complications among Adults with Diabetes in the United States
Source: J Diabetes Res. 2020 May 15;2020:2864069. doi: 10.1155/2020/2864069 (PMC7245693; doi:10.1155/2020/2864069)
Supplement: Supplementary Materials — Adjusted mean total direct medical expenditure for chronic kidney disease and eye complications (2015 US$). [file 2864069.f1.docx]

| **Appendix 1**  **Adjusted Mean Total Direct Medical Expenditure for Chronic Kidney Disease and Eye Complications (2015 US$)**  **(N=8,415)**  **Medical Expenditure Panel Survey (2009, 2011, 2013 and 2015)** | | | |
| --- | --- | --- | --- |
|  |  |  |  |
|  | Adjusted Mean | 95% CI | P-Value |
| No Eye Complications (ref) |  |  |  |
| Eye Complications | $3,325 | ($1,799 – $4,852) | <0.001 |
| No CKD (ref) |  |  |  |
| CKD | 7964 | (5332 – 10596) | <0.001 |
| **Sex** |  |  |  |
| Women (Ref) |  |  |  |
| Men | $725 | (-$347 – $1,798) | 0.184 |
| **Age** |  |  |  |
| 21-39 |  |  |  |
| 40-49 | -$585 | (-$3,354 – $2,185) | 0.678 |
| 50-64 | $820 | (-$1,723 – $3,364) | 0.526 |
| 65, + | $595 | (-$2,103 – $3,294) | 0.664 |
| **Race** |  |  |  |
| White (Ref) |  |  |  |
| African American | -$844 | (-2,124 – $436) | 0.195 |
| Hispanic | -2,267 | (-3,892 – -$642) | 0.006 |
| Other | -2,155 | (-4,467 – $157) | 0.067 |
| **Poverty Status** |  |  |  |
| Poor |  |  |  |
| Near Poor | -$1,839 | (-$3,854 – $177) | 0.074 |
| Middle Income | -$1,988 | (-$4,083 – $107) | 0.063 |
| High Income | -$1,984 | (-$4,317 – $349) | 0.095 |
| **Health insurance** |  |  |  |
| Private |  |  |  |
| Public | $18 | (-$1,632 – $1,669) | 0.983 |
| Uninsured | -$7,485 | (-$8,816 – -$6,154) | <0.001 |
| **Prescription Drug Coverage** | |  |  |
| Yes (ref) |  |  |  |
| No | -$1,929 | (-$3,424 – -$435) | 0.012 |
| **Perceived Physical Health** |  |  |  |
| Excellent/very good (ref) |  |  |  |
| Good | $2,229 | ($1,063 – $3,395) | <0.001 |
| Fair/Poor | $6,378 | ($4,812 – $7,944) | <0.001 |
| **Chronic physical conditions number** | |  |  |
| No Physical Condition (ref) |  |  |  |
| 1-2 | $5,038 | ($4,173 – $5,902) | <0.001 |
| 3-4 | $9,985 | ($8,637 – $11,333) | <0.001 |
| >=5 | $14,546 | ($12,240 – $16,852) | <0.001 |
| **Perceived mental health** |  |  |  |
| Excellent/very good (ref) |  |  |  |
| Good | -$603 | (-$1,680 – 473) | 0.27 |
| Fair/Poor | $1,118 | (-$418 – $2,655) | 0.153 |
| **Marital Status** |  |  |  |
| Married (ref) |  |  |  |
| Widow | $1,476 | (-$72 – $3,025) | 0.062 |
| Separated/Divorced | -$153 | (-$1,631 – $1,325) | 0.838 |
| Never Married | $420 | (-$1,127 – $1,967) | 0.593 |
| **Education** |  |  |  |
| Less than high school (ref) |  |  |  |
| High School | -$46 | (-$1,455 – $1,363) | 0.949 |
| >High School | $1,782 | ($226 – $3,337) | 0.025 |
| **Region of Residence** |  |  |  |
| Northeast |  |  |  |
| Midwest | 335 | (-1676 – 2345) | 0.743 |
| South | -1694 | (-3239 – -149) | 0.032 |
| West | -1843 | (-3533 – -154) | 0.033 |
| **Current Smoker** |  |  |  |
| Yes (ref) |  |  |  |
| No | $900 | (-$318 – $2,118) | 0.147 |
| **Heavy physical exercise** |  |  |  |
| Yes (ref) |  |  |  |
| No | $1,676 | ($595 – $2,757) | 0.003 |
|  |  |  |  |
